# Probable Catastrophic Antiphospholipid Syndrome Overlapping Hematological Abnormalities: A Case Report

**DOI:** 10.1002/ccr3.72718

**Published:** 2026-05-17

**Authors:** Faseeh Ullah, Maryam Tariq, Marwa Farzand, Irha Riaz, Rizwan Rasol Khan, Kritick Bhandari

**Affiliations:** ^1^ Aziz Fatimah Medical and Dental College Faisalabad Pakistan; ^2^ Aziz Fatimah Medical and Dental College–Aziz Fatimah Hospital Faisalabad Pakistan; ^3^ KIST Medical College and Teaching Hospital Lalitpur Nepal

**Keywords:** anemia, antiphospholipid syndrome, autoimmune, hemolytic, plasmapheresis, thrombocytopenia

## Abstract

A young South Asian woman presented four weeks post‐miscarriage with fever, jaundice, edema, headache, neurological deficits, anemia, thrombocytopenia, proteinuria, hematuria, splenic infarct, and cerebral venous sinus thrombosis. Positive beta‐2 glycoprotein IgG and lupus anticoagulant supported probable CAPS. Transfusions and plasmapheresis resulted in significant clinical improvement.

## 1 Introduction

Antiphospholipid syndrome (APS) is a systemic autoimmune disorder characterized by the occurrence of vascular thrombosis and obstetric complications [1]. Affecting approximately 1% of individuals with APS or 5/million in the general population across the world, Catastrophic Antiphospholipid Syndrome (CAPS) is a rare presentation of APS, debilitating a patient's life [2]. It is characterized by multiple vascular occlusive events involving small vessels, over a short period, confirmed by histopathology, and the presence of autoantibodies [3]. Hematological abnormalities like autoimmune hemolytic anemia (AIHA) and thrombocytopenia, although not included in clinical criteria, are often co‐occurring with antiphospholipid antibodies [4]. The following case highlights the coexistence of hematological variations and CAPS. The case is being reported after taking written informed consent in national language from the patient and her family. The case report has been formulated as per standard The CAREGuidelines: Consensus‐based Clinical Case Reporting Guideline Development [5].

## 2 Case History/Examination

A 25‐year‐old married Asian female presented to the emergency room (ER) with a fever for 3 weeks, a headache for 2 days, and vomiting for a day. She had high‐grade fever (102°F), accompanied by chills, generalized weakness, and a sudden, severe occipital headache that radiated to the shoulders and back, associated with blurred vision. She had 10–12 episodes of vomiting/day, devoid of blood. She was married for 4 years and had a dilatation and curettage (D&C) a month back at 24 weeks of gestation. She had a history of two first‐trimester pregnancy losses and shared a similar family history of recurrent miscarriages.

**FIGURE 1:**
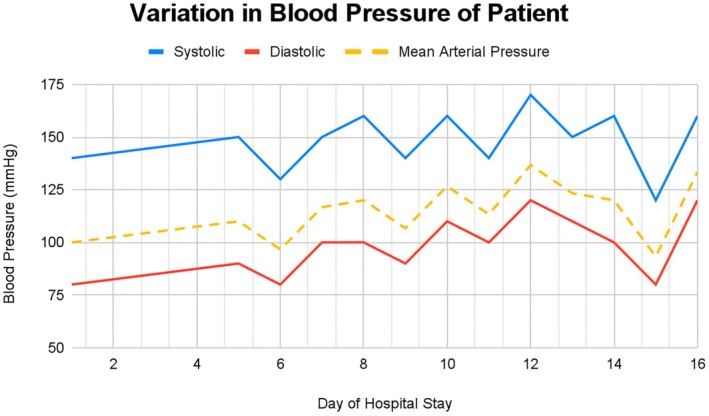
Variation in the patient's blood pressure on subsequent days of hospital stay.

Upon presentation, she had pedal edema, pallor, and jaundice, though vitally stable except for blood pressure (B.P.) (Figure 1). She was conscious and well oriented (GCS 15/15) with right 6th nerve palsy and ipsilateral body weakness (power of 2/5 in right upper limb and 4/5 in right lower limb) with downgoing plantar reflex. There were no signs of meningeal irritation.

## 3 Differential Diagnosis, Investigations and Treatment

The pre‐admission workup revealed thrombocytopenia and life‐threatening anemia, characterized by polychromasia and increased RBC indices (MCV, MCH) on the peripheral smear. An ultrasound abdomen‐pelvis two days before admission demonstrated a normal‐sized liver with mild parenchymal echogenicity and minimal splenomegaly containing multiple irregular hypoechoic lesions.

Upon admission, her labs showed a positive Coombs test, an elevated reticulocyte count (6%), and Lactate Dehydrogenase (1379 U/L) with a normal clotting profile and electrolytes. (Day 01 of Admission) A triphasic CT abdomen requested on the admission day resonated with pre‐admission ultrasound, with the addition of hepatomegaly and splenomegaly with evidence of splenic infarcts. Symptomatic treatment comprising of fluids, anti‐emetics, analgesics, and anticoagulants was started. Differential diagnosis of catastrophic antiphospholipid syndrome (CAPS), thrombotic thrombocytopenic purpura (TTP), autoimmune hemolytic anemia (AIHA), and Systemic Lupus Erythematosus (SLE) with secondary APS were suspected.

The next day, her GCS dropped to 12/15 (Day 02 of admission), and D‐dimers were raised. Intraocular pressure monitoring was started due to raised intracranial pressure. Upon consultation with the neurologist, CT arteriography and venography of the brain were ordered to rule out thrombosis in the brain, especially cerebral venous sinus thrombosis (CVST). Moreover, echocardiography ruled out the presence of any clots in the cardiac chamber. Furthermore, packed red blood cells (03 units) and platelets (01 unit) were also transfused.

**FIGURE 2:**
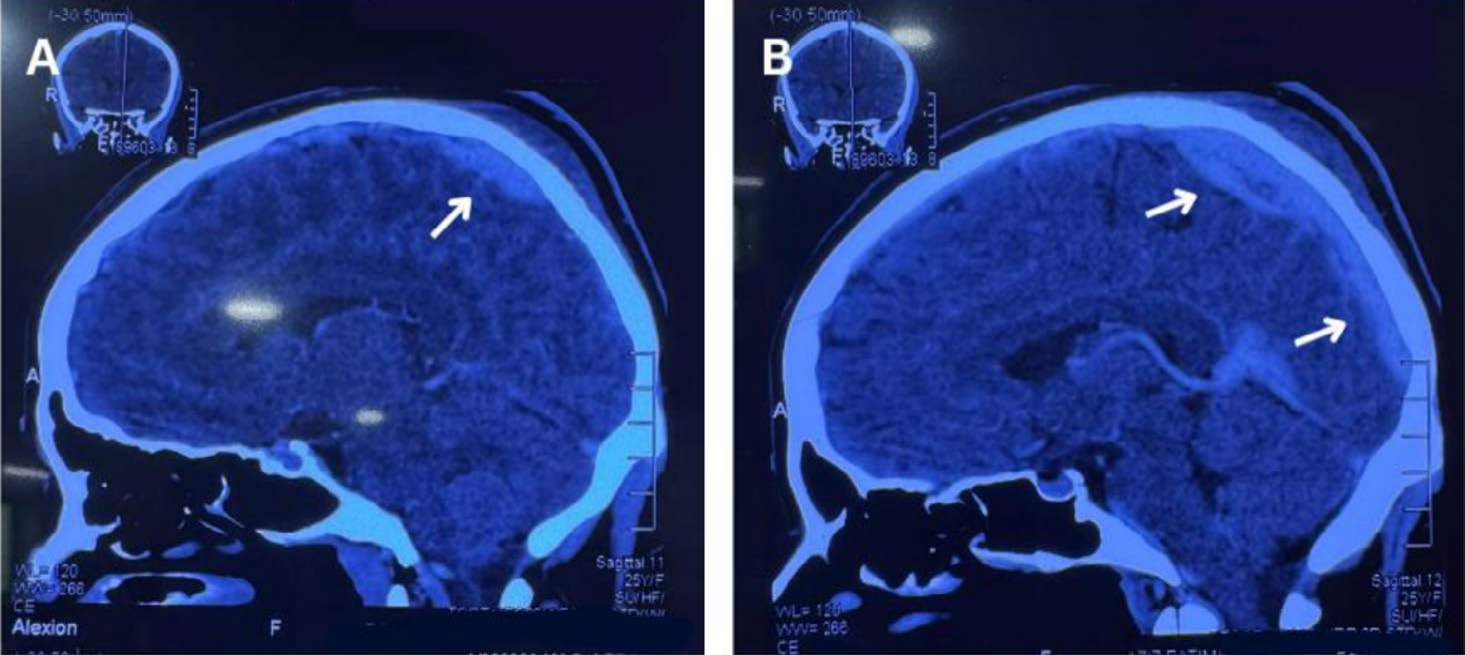
CT‐Venography showing Cerebral Venous Sinus Thrombosis (A) with involvement of the Superior sagittal and left transverse venous sinuses (Arrows) (B).

An autoimmune etiology was suspected; therefore, an autoimmune profile was requested, which ruled out hepatitic and systemic autoimmune disorders like SLE by the 5th day of hospital stay. (Table 1) Imaging confirmed cerebral venous sinus thrombosis (CVST) as shown in Figure 2 (Day 05 of admission). On Day 7, CBC continued to show thrombocytopenia and macrocytic anemia with neutrophil predominance, lymphopenia, and eosinopenia (Table 2). Sodium bicarbonate levels were elevated, and pedal edema had resolved. Lupus anticoagulant (HC23) was positive, and by the following day, β2‐glycoprotein IgG antibodies (45 RU/mL) were also detected. Hence, based on these findings, a diagnosis of Suspected Catastrophic Antiphospholipid Syndrome (CAPS) associated with Autoimmune Hemolytic Anemia (AIHA) was established, while TTP was ruled out (Day 08 of admission).

**TABLE 1:** Autoimmune profile of the patient as per differentials being considered.

Disease/Target	Test	Result
Systemic Lupus Erythematosus (SLE)	ANA (antinuclear antibodies)	Negative
	Anti‐dsDNA antibodies	Negative
	Anti‐Nucleosome antibodies	Negative
	Anti‐Histone antibodies	Negative
Antiphospholipid Syndrome (APS)	HC23‐Lupus Anticoagulant	Positive
	Anti‐β2 Glycoprotein 1 (anti‐B2GP1) antibodies	Positive
Autoimmune Hepatitis/Liver Disease	ASMA (anti‐smooth muscle antibodies)	Negative
	AMA (anti‐mitochondrial antibodies)	Negative
	LKM (liver‐kidney microsomal antibodies)	Negative
Gastric Autoimmunity	GPC (gastric parietal cell antibodies)	Negative

**TABLE 2:** Complete blood count of the patient during her illness.

Test		Days of Hospital Stay		
	Pre‐admission	7th day	8th day	10th day
WBC (4–11 × 10^3^/uL)	9.95	9.81	5.12	7.6
RBC (2.5–5.5 million/mL)	1.81	3.71	3.82	3.75
Hb (female: 12–14 g/dL)	5.9	11.4	11.7	11.6
HCT (40%–52%)	18.9	36.4	36.9	36.5
MCV (79–95 fl)	104.4	98.1	96.6	97.3
MCH (26–32 pg)	32.6	30.7	30.6	30.9
MCHC (30–36 g/dL)	31.2	31.3	31.7	31.8
Platelet count (140–450 10^3^ u/L)	30	101	141	195
Neutrophils (50%–70%)	50	84	82	76
Lymphocytes (25%–40%)	40	10	12	14
Monocytes (2%–11%)	6	5	6	10
Eosinophils (2%–6%)	4	1	0	0

Upon neurology consultation, the patient had the first session of plasmapheresis, which was conducted on day‐12 of admission. The very next day, motor power improved. One unit of Fresh Frozen Plasma (FFP) was transfused. The next day, the patient was discharged after the third session of plasmapheresis. On the discharge slip, the patient was prescribed heparin as an anticoagulant for 3–5 days. Following that, the patient was put on lifetime anticoagulation with warfarin.

## 4 Conclusion and Results (Outcome and Follow‐Up)

Furthermore, two sessions of plasmapheresis were given during the first week post‐discharge. Her condition, including motor strength and CN‐VI palsy, improved. Further follow‐up was lost.

## 5 Discussion

The case involves a young female with clinical characteristics of coexisting probable CAPS and AIHA, which is rare and poses a diagnostic and treatment challenge, with a paradoxical tendency of both thrombosis and bleeding in the patient. A meta‐analysis reported that the pooled prevalence of AIHA was significantly higher in patients with SLE‐related APS (22.8%) than in those with primary APS (PAPS) (3.9%), indicating that AIHA occurs predominantly in the context of secondary APS [6]. Furthermore, the prevalence of the Direct anti‐globulin test (DAT) was also higher in SLE patients with aPL (37.7%) than in PAPS (16%), therefore indicating that the coexistence of SLE and aPL confers a greater risk for autoimmune hemolysis compared to PAPS alone [6]. In contrast, the current case lacked a history of SLE and was newly diagnosed with PAPS complicated by CAPS and AIHA, an unusual presentation.

Our patients tested positive for Lupus anticoagulant and anti‐B2GP1 antibodies, strongly supporting the diagnosis of APS. Declaring definitive CAPS requires four of the following: involvement of three or more organs, clinical manifestations of the disease within less than a week, histopathological evidence of thrombosis, and positive APL antibodies [7]. The limitation of this case is that histopathological evidence was not obtained due to the patient's critical condition; therefore, our case met three out of the four criteria, fulfilling the definition of probable CAPS. Infectious causes were considered unlikely given a normal WBC count, while the acute clinical onset and negative Nucleosome antibodies, dsDNA antibodies, and Histone antibodies effectively ruled out SLE. Thrombocytopenia (platelet count: 30 × 10^3^ u/L) was reported in our patient, likely resulting from immune‐mediated platelet destruction by antigen–antibody complex and splenic sequestration secondary to splenomegaly [8]. Clinical manifestations like thrombocytopenia, blurred vision, and raised retic count initially raised the suspicion of TTP [9] although this was not confirmed by ADAMTS13 activity. However, immediate plasma exchange was deferred because imaging was consistent with splenic infarcts and CVST, together with a history of recurrent pregnancy losses, favored the diagnosis of CAPS over TTP, where plasmapheresis alone may worsen the thrombosis. In addition, positive DA, along with elevated LDH and reticulocytosis, led to the diagnosis of autoimmune hemolytic anemia [10]. This case highlights a rare coexistence of probable CAPS with AIHA, contributing to valuable insights to the limited literature on overlapping autoimmune and thrombotic disorders.

CAPS is an uncommon but fulminant manifestation of APS characterized by rapid onset microvascular thrombosis involving multiple organs: nervous, renal, pulmonary, enteral, and cutaneous, with approximately 69% of cases reported in women [11]. The usual manifestations of CAPS include renal failure (77%) and arterial hypertension (27%), followed by altered consciousness (39%), acute respiratory syndrome (36%), and splenic infarcts (18%) [12]. Likewise, in our patient, nervous, splenic, and renal manifestations were evident by clinical features and lab reports. The acute and fulminant nature of CAPS allows microvascular thrombosis to produce rapid changes in organ size, particularly in the spleen and liver, as observed on imaging in our patient. The triggering factors of CAPS are infections (27%), pregnancy (22%), surgery (15%), lupus flares (7%), and malignancy (2%) [13]. Our patient had a D&C at 24 weeks, suggesting pregnancy as the primary precipitating factor.

Upon suspicion of CAPS, prompt initiation of triple therapy, which consists of anticoagulants, corticosteroids, and plasma exchange, is recommended to reduce mortality by 37% [3]. The thrombotic state, including CVST, as observed in our patient, was treated initially with anticoagulants, followed by steroids. The major challenge was to treat thrombocytopenia due to AIHA and CAPS‐induced hypercoagulable state simultaneously. Hence, the platelet transfusions were carried out carefully, maintaining the balance, given the potential risk of exacerbating thrombosis. Five sessions of plasmapheresis were done, resulting in rapid and notable improvement in her neurological status, likely reflecting the procedure's effectiveness in removing circulating APL antibodies and complement factors and halting ongoing microvascular thrombosis [14]. Subsequently, the patient has been transitioned from heparin to lifetime anticoagulation with warfarin.

CAPS can be the initial presentation of APS, and differentiating it from other thrombotic microangiopathies like TTP or DIC is a challenge due to overlapping features. Early diagnosis and prompt treatment are crucial in such patients; thus, this case highlights the need to consider CAPS as a potential cause in patients with rapidly progressing thrombotic events, particularly when multiple organs are involved.

## Author Contributions

**Faseeh Ullah:** conceptualization, data curation, formal analysis, investigation, methodology, project administration, validation, visualization, writing – original draft, writing – review and editing. **Maryam Tariq:** data curation, formal analysis, investigation, methodology, visualization, writing – original draft, writing – review and editing. **Marwa Farzand:** data curation, methodology, supervision, validation, writing – original draft, writing – review and editing. **Irha Riaz:** formal analysis, validation, writing – original draft, writing – review and editing. **Rizwan Rasol Khan:** conceptualization, investigation, methodology, validation, writing – original draft, writing – review and editing. **Kritick Bhandari:** data curation, formal analysis, supervision, writing – original draft, writing – review and editing.

## Funding

The authors have nothing to report.

## Ethics Statement

The authors have nothing to report.

## Consent

Written informed consent was obtained from the patient and her family for publication of this case report and accompanying images.

## Conflicts of Interest

The authors declare no conflicts of interest.

## Data Availability

Data sharing not applicable – no new data generated, or the article describes entirely theoretical research.
